# Early electroacupuncture treatment ameliorates neuroinflammation in rats with traumatic brain injury

**DOI:** 10.1186/s12906-016-1457-6

**Published:** 2016-11-16

**Authors:** Wei-Chen Tang, Yao-Chin Hsu, Che-Chuan Wang, Chiao-Ya Hu, Chung-Ching Chio, Jinn-Rung Kuo

**Affiliations:** 1 Department of Chinese Medicine, Tainan Municipal An-Nan Hospital, Tainan, Taiwan; 2 Department of Chinese Medicine, Chi-Mei Medical Center, Tainan, Taiwan; 3 Department of Neurosurgery, Chi-Mei Medical Center, Tainan, Taiwan; 4 Department of Medical research, Chi-Mei Medical Center, Tainan, Taiwan; 5 Department of Child Care, Southern Taiwan University of Science and Technology, Tainan, Taiwan; 6 Department of Biotechnology, Southern Taiwan University of Science and Technology, Tainan, Taiwan; 7 Traumatic Brain Injury Center, Chi Mei Hospital, No. 901, Zhonghua Rd., Yongkang Dist., Tainan City, 710 Taiwan, ROC

**Keywords:** Astrocyte, Electroacupuncture, Microglia, Neuronal apoptosis, Traumatic brain injury, Tumor Necrosis factor-α

## Abstract

**Background:**

Neuroinflammation is the leading cause of neurological sequelae after traumatic brain injury (TBI). The aim of the present study was to investigate whether the neuroprotective effects of electroacupuncture (EA) are mediated by anti-neuroinflammatory effects in a rat model of TBI.

**Methods:**

Male Sprague-Dawley rats were randomly divided into three groups: sham-operated, TBI control, and EA-treated. The animals in the sham-operated group underwent a sham operation, those in the TBI control group were subjected to TBI, but not EA, and those in the EA group were treated with EA for 60 min immediately after TBI, daily for 3 consecutive days. EA was applied at the acupuncture points GV20, GV26, LI4, and KI1, using a dense-dispersed wave, at frequencies of 0.2 and 1 Hz, and an amplitude of 1 mA. Cell infarction volume (TTC stain), neuronal apoptosis (markers: TUNEL and Caspase-3), activation of microglia (marker: Iba1) and astrocytes (marker: GFAP), and tumor necrosis factor (TNF)-α expression in the microglia and astrocytes were evaluated by immunofluorescence. Functional outcomes were assessed using the inclined plane test. All tests were performed 72 h after TBI.

**Results:**

We found that TBI-induced loss of grasp strength, infarction volume, neuronal apoptosis, microglial and astrocyte activation, and TNF-α expression in activated microglia and astrocytes were significantly attenuated by EA treatment.

**Conclusions:**

Treatment of TBI in the acute stage with EA for 60 min daily for 3 days could ameliorate neuroinflammation. This may thus represent a mechanism by which functional recovery can occur after TBI.

## Background

Neuroinflammation is the major cause of disability and death after traumatic brain injury (TBI). Activated astrocytes and microglia are markers of neuroinflammation after TBI [1, 2]. These activated cells can release tumor necrosis factor-alpha (TNF-α) and can signal neuronal apoptosis and impair brain function [3, 4]. Therefore, attenuating reactive microgliosis and astrogliosis may be a promising strategy for the treatment of the neurological sequelae of TBI.

Electroacupuncture (EA), a highly popular traditional Chinese therapy, is also widely used in the USA, with 2.1 million adults undergoing EA per year [5]. Previous studies have demonstrated the beneficial effects of EA on stroke [6–8], spinal cord injury [9], arthritis [10], and sciatica [11]. Recently, we have demonstrated that application of EA 60 min post-TBI has neuroprotective effects on neuronal cells. These effects might be attributable to the anti-apoptotic effects of EA, as demonstrated in the injured cortex in a fluid-percussion model of TBI [12]. However, the effects of EA on neuroinflammation after TBI still require clarification.

In this study, we tested the hypothesis that EA therapy attenuates TBI-induced cerebral injury and improves neurological outcomes by inhibiting activation of microglia and astrocytes, as well as TNF-α expression in activated microglia and astrocytes, after TBI. To this end, we assessed neuronal apoptosis and TNF-α expression in activated microglia and astrocytes in the ischemic cortex at 72 h after TBI. We also compared motor deficits and cerebral infarction volume after TBI in rats that did or did not receive EA therapy for 60 min per day for 3 days.

## Methods

### Animals

Adult male Sprague-Dawley (SD) rats, weighing 360 ± 20 g, were used in these experiments. All experimental procedures conformed to the NIH guidelines and were approved by the Institutional Animal Care and Use Committee (IACUC) of Chi Mei Medical Center (IACUC Approval NO 10012722). Care was taken to minimize discomfort of the animals during surgery and the recovery period. At the end of the experiments, the rats were sacrificed with an overdose of urethane.

### Traumatic brain injury

Animals were anesthetized by intramuscular administration of a mixture of ketamine (44 mg/Kg, Nankuang Pharmaceutical, Tainan, Taiwan), atropine (0.02633 mg/kg, Sintong Chemical Ind. Co., Taoyuan, Taiwan), and xylazine (6.77 mg/kg, Bayer, Berlin, Germany). Using a stereotaxic frame, a craniectomy defect with a 2-mm radius was created in the right parietal cortex. Then, a fluid percussion device (VCU Biomedical Engineering, Richmond, VA, USA) was connected, and the brain was injured with a 2.0–2.2 atm, 25-ms percussion. This method produces moderately severe brain trauma, as described by Mclntosh et al. [13]. Detailed procedures are previously described [12].

### Treatment intervention

The rats were randomly divided into three groups: Sham operation, TBI control, and EA treatment immediately after TBI. EA was applied at the acupuncture points Baihui (GV20), Shuigou (GV26), Hegu (LI4), and Yongquan (KI1) (WHO standard names), using a dense-dispersed wave at frequencies of 0.2 and 1 Hz, and an amplitude of 1 mA (low frequency 5-channel TENS Unit and Electrical Needle Stimulator, model 05B, Ching Ming Medical Device Co., Ltd., Taipei, Taiwan) for 60 min per day, for 3 days. For each group of measurement parameters, we used six rats. The experimental endpoint was measured 3 days after TBI as lateral fluid percussion causes motor dysfunction from 3 days to 1 year after TBI [14].

### Cerebral infarction assay

The infarction volume was measured using triphenyltetrazolium chloride (TTC) staining at 72 h after TBI. TTC staining was performed as described previously [15]. Under deep anesthesia (sodium pentobarbital, 100 mg/kg, i.p.), the animals were administered an intracardiac perfusion of saline. The brain tissue was then removed, immersed in cold saline for 5 min, and sliced into 2.0-mm-thick sections. The brain slices were incubated in 2% TTC dissolved in phosphate-buffered saline (PBS) for 30 min at 37 °C, and then fixed in 5% formaldehyde solution. The infarction volume, as revealed by negative TTC stains, indicating dehydrogenase-deficient tissue, was measured in each slice and summed using computerized planimetry (Media Cybernetics, Inc. Washington Street, Rockville, USA). The infarction volume was calculated as 2 mm (thickness of the slice) × [the sum of the infarction areas in all brain slices (mm^2^)].

### Motor function evaluation

An inclined plane was used to measure limb strength. The animals were placed facing right and then facing left, perpendicular to the slope of a plane inclined at 55° (20 cm × 20 cm buffer-ribbed surface) [16]. To determine the maximum angle at which an animal could remain on the inclined plane, the angle was increased or decreased in increments of 5°. Motor deficits were measured at the left- and right-side maximal angles, at 72 h after TBI.

### Immunofluorescence assays

At 72 h after TBI, consecutive 6-μm thick sections, corresponding to coronal coordinates 2.0–7.0 mm posterior to the bregma, were obtained as described previously [17]. Activated microglia and astrocytes were evaluated by detecting Iba1- and GFAP-positive cells, respectively, using an immunofluorescence assay [18]. TNF-α expression in the activated microglia and astrocytes was investigated by detecting Iba1/GFAP plus TNF-α-positive cells using an immunofluorescence assay. Apoptotic neuronal cells were identified by double-staining with terminal deoxynucleotidyltransferase-mediated dUTP-biotin nick-end labeling (TUNEL) or Caspase-3 and Neu-N staining [19]. The following antibodies were used: a monoclonal mouse anti-Iba1 antibody (ab1211, Abcam, Boston, MA, USA) at a 1:400 dilution, detected with a DyLight® 594 anti-mouse (IgG) antibody (ab96873, Abcam) at a 1:400 dilution; a polyclonal rabbit anti-TNF-α antibody (ab6671, Abcam) at a 1:200 dilution, detected with an Alexa-Fluor® 488 anti-rabbit (IgG) antibody (ab150063, Abcam) at a 1:1000 dilution; a monoclonal mouse anti-NeuN antibody (ab104224, Abcam) at a 1:1000 dilution, detected with a DyLight® 594 anti-mouse (IgG) antibody (ab96873, Abcam) at a 1:400 dilution; a monoclonal mouse anti-GFAP antibody (ab10062, Abcam) at a 1:1000 dilution, detected with a DyLight® 594 anti-mouse (IgG) antibody (ab96873, Abcam) at a 1:400 dilution; a monoclonal rabbit anti-Caspase-3 antibody (#9664, Cell Signaling Technology, Beverly, MA, USA) at a 1:400 dilution, detected with an Alexa Fluor® 488 anti-rabbit (IgG) antibody (ab150073, Abcam) at a 1:1000 dilution. The number of positively stained cells was calculated in five coronal sections corresponding to peri-lesional of ipsilateral cortex (original magnification, 400 × (10 × 40)) and expressed as the mean number of positive cells in all five sections from each rat using computerized planimetry (Image-Pro Plus Media Cybernetics, Inc., Rockville, MS, USA).

### Statistical analysis

The results are expressed as the means ± standard deviation. A two-way analysis of variance for repeated measurements was used for factorial experiments, and Dunnett’s test was used for post hoc multiple comparisons among means. Differences were considered significant at *p* < 0.05. All data were analyzed with Sigma Plot version 11.0 for Windows (Systat Software, San Jose, CA, USA).

## Results

### Effects of EA on functional outcome measures assessed on the inclined plane

**Fig. 1:**
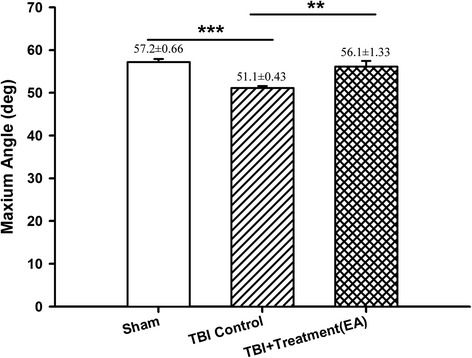
The effects of electroacupuncture (EA) treatment on traumatic brain injury (TBI)-induced motor deficits, as investigated using the inclined plane test to determine the maximum grasp angle at 72 h after TBI. ***p < 0.001compared with the sham group; **p < 0.01 compared with EA treatment in the TBI group, *n* = 6 in each group

The maximal grip angle of rats at 72 h after TBI was significantly lower than that of the sham controls (51.1° ± 0.43° versus 57.2° ± 0.66°, respectively, *p* < 0.001). The TBI-induced motor dysfunction was significantly improved by EA treatment (TBI group versus EA group, 51.1° ± 0.43° versus 56.1° ± 1.33°, *p* < 0.01; Fig. 1).

### EA significantly decreases TBI-induced cerebral infarction volume

**Fig. 2:**
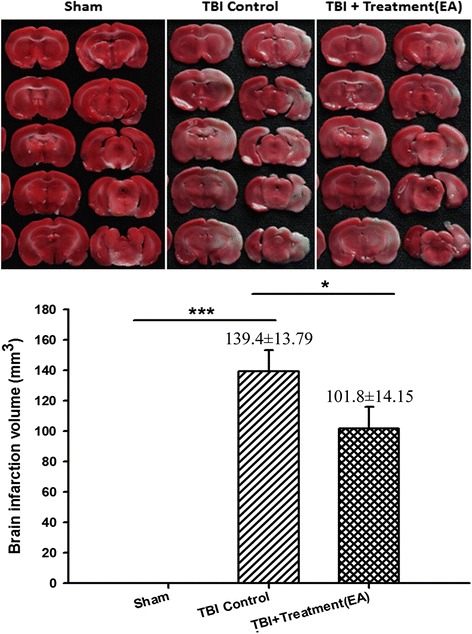
The effects of electroacupuncture (EA) on traumatic brain injury (TBI)-induced infarction volume in the ischemic cortex at 72 h after TBI, ***p < 0.001 compared with the sham group; *p < 0.05 compared with EA treatment in the TBI group, *n* = 6 in each group

At 72 h following TBI, the TTC-stained volume was significantly higher in the infracted area of TBI controls than in the corresponding area of the sham controls (139.4 ± 13.8 mm^3^ versus 0, *p* < 0.001; *n* = 6 per group). The TBI-induced infarction volume was significantly decreased by EA treatment (TBI group versus EA group, 139.4 ± 13.8 mm^3^ versus 101.8 ± 14.2 mm^3^, *p* < 0.05; *n* = 6 per group; Fig. 2).

### EA decreases neuronal apoptosis in the peri-lesional cortex after TBI

**Fig. 3:**
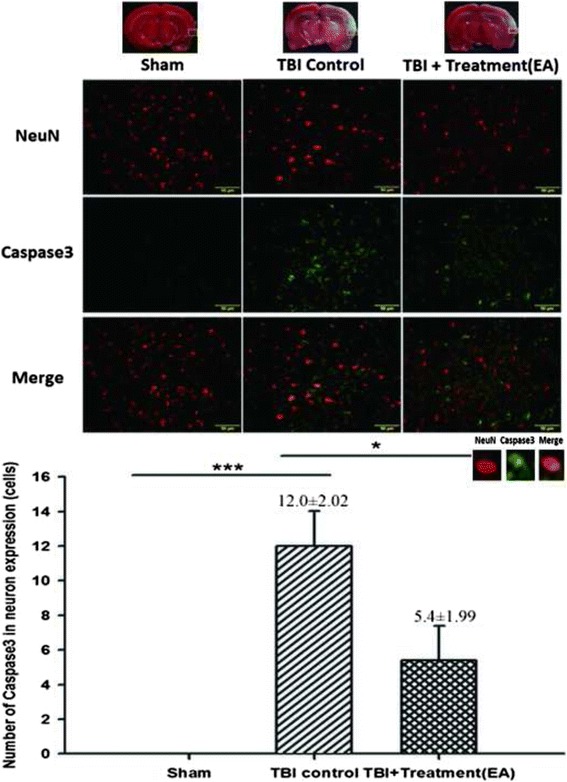
The effects of electroacupuncture (EA) treatment on Caspase-3 expression in cortical neurons (markers: Caspase-3 plus Neu-N) at 72 h after traumatic brain injury (TBI), ***p < 0.001, compared with the sham group; *p < 0.05 compared with EA treatment in the TBI group, *n* = 6 in each group. The areas measured for TTC staining are marked with squares in the top panel

First, using the Caspase-3 assay, we found that the number of Caspase-3 expressing neurons (i.e., double-positive for Neu-N and Caspase-3) in the peri-lesion cortex was significantly higher in the TBI group at 72 h after TBI than in the sham controls (12.0 ± 2.02 and 0 ± 0, respectively; *p < 0.001*; *n* = 6 both groups). However, this number was significantly reduced after EA treatment (TBI group versus EA group, 12.0 ± 2.02 versus 5.4 ± 1.99; *p <* 0.05; *n* = 6 both groups; Fig. 3).

**Fig. 4:**
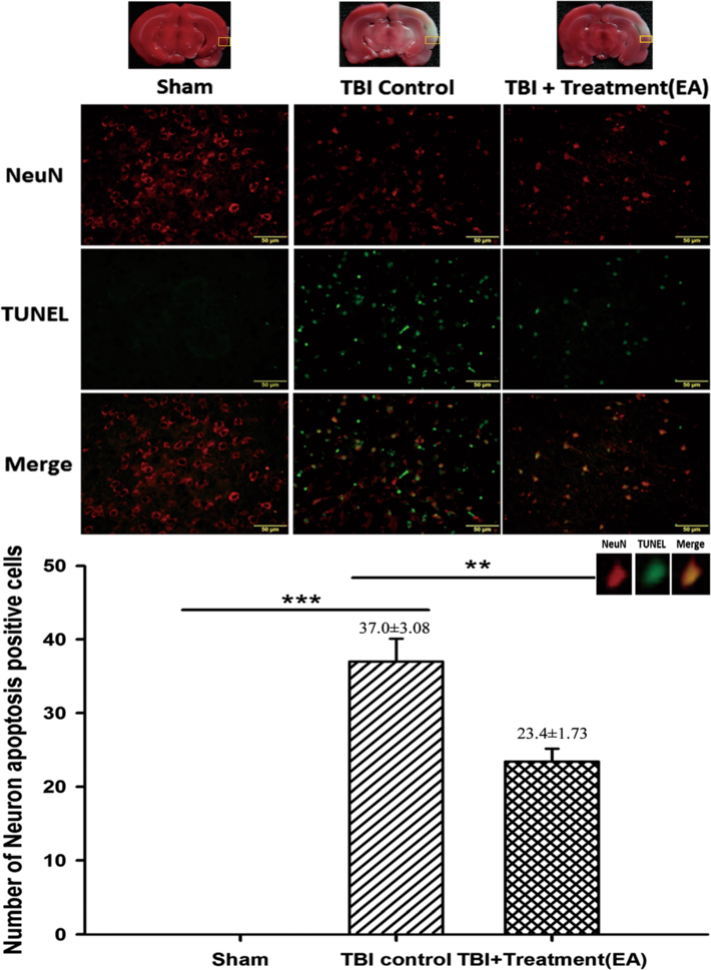
The effects of electroacupuncture (EA) treatment on traumatic brain injury (TBI)-induced neuronal apoptosis (markers: Neu-N plus TUNEL) at 72 h after TBI, ***p < 0.001 compared with the sham group; **p < 0.01 compared with EA treatment in the TBI group, *n* = 6 in each group. The areas measured for TTC staining are marked with squares in the top panel

Then, using the TUNEL assay, we found that the number of apoptotic neuronal cells (positive for Neu-N plus TUNEL staining) in the peri-lesional cortex was significantly higher in the TBI group at 72 h after TBI than in the sham controls (37.0 ± 3.08 and 0 ± 0, respectively; *p* < 0.001; *n* = 6 both groups). Moreover, this number was significantly reduced after EA treatment (TBI group versus EA group, 37.0 ± 3.08 versus 23.4 ± 1.73; *p <* 0.01; *n* = 6 both groups; Fig. 4).

### EA attenuates activation of microglia and astrocytes in the peri-lesional cortex

**Fig. 5:**
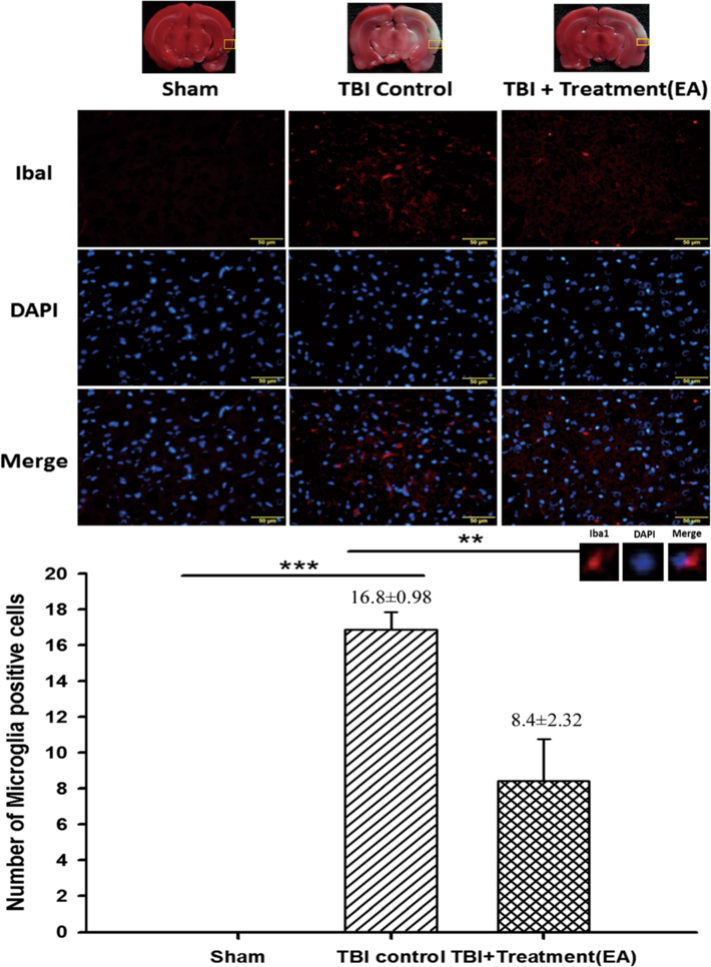
The effects of electroacupuncture (EA) treatment on the activation of microglia in the ischemic cortex (markers: Iba1 plus DAPI) at 72 h after traumatic brain injury (TBI). ***p < 0.001 compared with the sham group; **p < 0.01 compared with EA treatment in the TBI group, *n* = 6 in each group. The areas measured for TTC staining are marked with squares in the top panel

**Fig. 6:**
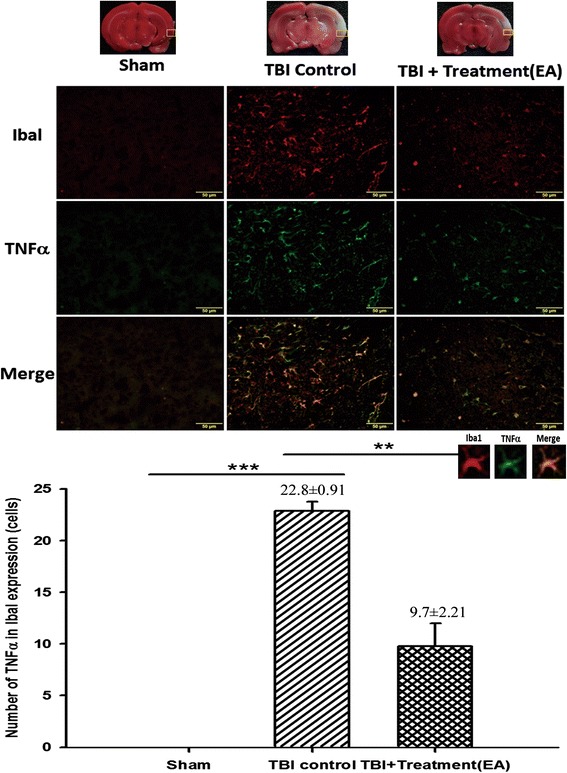
The effects of electroacupuncture (EA) treatment on activation of cortical astrocytes (markers: GFAP plus DAPI) at 72 h after traumatic brain injury (TBI), ***p < 0.001 compared with the sham group; **p < 0.05 compared with EA treatment in the TBI group, *n* = 6 in each group. The areas measured for TTC staining are marked with squares in the top panel

We evaluated microglial and astroglial activation and tested the possibility that EA might suppress TBI-induced brain microgliosis and astrogliosis. Microgliosis was represented by microglia with an amoeboid morphology, with retracted, thickened processes and an enlarged soma. Iba1-DAPI and GFAP-DAPI double-staining showed that the number of microglia was significantly increased in the peri-lesional cortex of the TBI rats than in the sham rats. EA significantly attenuated this TBI-induced activation of microglia (*p* < 0.01, Fig. 5) and astrocytes (*p* < 0.01, Fig. 6).

### EA attenuates TNF-α expression in activated microglia and astrocytes in the peri-lesional cortex

**Fig. 7:**
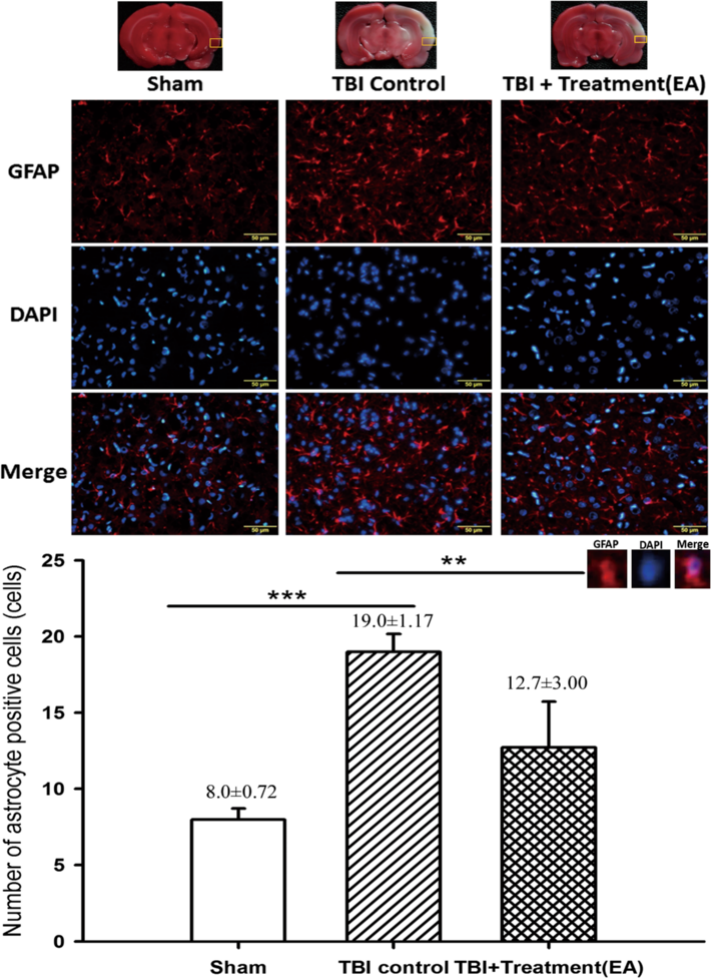
The effects of electroacupuncture (EA) treatment on tumor necrosis factor (TNF)-α expression in activated microglia in the ischemic cortex (markers: Iba1 plus TNF-α) at 72 h after traumatic brain injury (TBI), ***p < 0.001 compared with the sham group; **p < 0.01 compared with EA treatment in the TBI group, *n* = 6 in each group. The areas measured for TTC staining are marked with squares in the top panel

**Fig. 8:**
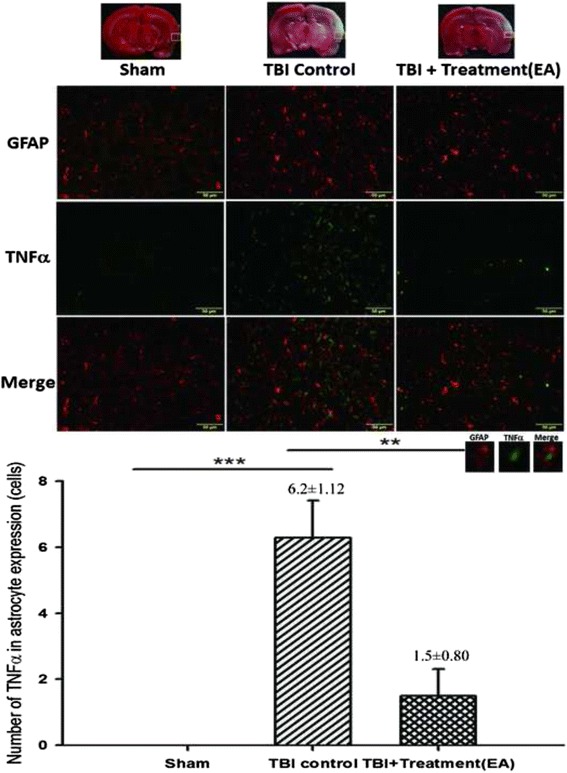
The effects of electroacupuncture (EA) treatment on tumor necrosis factor (TNF)-α expression in cortical astrocytes (markers: GFAP plus TNF-α) at 72 h after traumatic brain injury (TBI), ***p < 0.001 compared with the sham group; **p < 0.01 compared with EA treatment in the TBI group, *n* = 6 in each group. The areas measured for TTC staining are marked with squares in the top panel

We evaluated TNF-α expression in activated microglia and tested the possibility that EA might attenuate TBI-induced neuroinflammation. As predicted, the expression of Iba1 plus TNF-α and GFAP plus TNF-α in the peri-lesioned cortex was significantly higher in the TBI rats than in the sham rats. However, EA significantly reduced the TBI-induced TNF-α expression in activated microglia (*p* < 0.01, Fig. 7) and astrocytes (*p* < 0.01, Fig. 8).

## Discussion

In the current study, treatment of TBI with EA for 60 min per day for 3 days, using low frequencies of 0.2 and 1 Hz and an intensity of 1 mA, during the acute injury phase, was shown to decrease neuroinflammation and the expression of factors associated with neuronal apoptosis. This may represent a mechanism by which functional recovery may occur after TBI.

Most acupuncture-related research in Chinese medicine employs “acupoint groups”, which comprise two or more acupoints. Therefore, the therapeutic roles and mechanisms of single, specific acupoints are difficult to discern in these studies. Rather, the majority of acupuncture experimental research describes the synergistic effects of “acupoints groups”. Xu et al. investigated the effects of acupuncture at the acupoints Baihui (GV 20) and Zusanli (ST36) in an ischemia − reperfusion injury model after middle cerebral artery occlusion. They found that TNF-α expression was lower in the EA group than in the model and sham-operated groups [7]. In a study by Cheng et al., acupuncture at the Baihui (GV 20) and Dazhui (GV14) acupoints significantly downregulated the expression of TNF-α, GFAP, S100B, and nuclear factor-kB in the ischemic cortical penumbra [8]. Jiang et al. selected Shuigou (GV 26) and Fengfu (DU16) for acupuncture treatment of traumatic spinal cord injury and found that EA had anti-oxidative, anti-inflammatory, and anti-apoptotic effects as indicated by reduced expression of inflammatory cytokines, including TNF-*α* [9]. Gu et al. treated patients that had undergone laparoscopic cholecystectomy (LC) at Hegu (LI 4), Neiguan (PC6), Zusanli (ST 36), and Yanglingquan (GB 34), and found that the TNF-α levels decreased significantly at 3 days after LC [20]. In the current study, EA was applied at the acupuncture points GV20, GV26, LI4, and KI1; we found that this significantly attenuated neuroinflammation in a TBI model.

Related studies on EA therapy have employed different EA parameters, including EA frequency, waveform, and intensity. Liu et al. have reported that EA at a frequency of 2 and 5 Hz, 0.4–10 mA, with an intermittent waveform, was more effective for treatment of sciatica [11]. Chan et al. previously reported that EA at 2 Hz (low frequency) can provide neuroprotection by preserving retinal function in glaucomatous rats [21]. Kuai et al. compared the effects of EA between different waveforms (continuous, intermittent, and sound-electric waves); EA treatment of arthritis with intermittent waves increased the β-endorphin content in tissues with local inflammation [10]. Chuang et al. demonstrated that 60 min of EA treatment in the acute stage of TBI could show a better outcome than a 30-min treatment, as determine from an increase in the regional blood flow and attenuation of neuroinflammation-associated parameters [12].

In the current study, EA with sparse-dense wave of low frequency (0.2 Hz/1 Hz) and intensity of 1 mA was applied for 60 min daily for 3 days. Therefore, the therapeutic time used was 2–3 times that used in previous studies. This design was consistent with that used by Gu et al. [20], who used the same sparse-dispersed wave. Results of both studies showed that TNF-α levels were decreased in the injured tissues after EA treatment. In future, the efficacy of intermittent and sparse-dense waveforms should be compared, and the correlation between TNF-α levels and different EA waveforms should be investigated.

The timeline of TNF-α release varies, ranging from 1 h to months after TBI [22, 23]. Our findings on TNF-α expression and neuroinflammation at 72 h after TBI are in line with many previous results. TNF-α expression was significantly higher in the lesion boundary zone in TBI-control rats at 72 h post-TBI than in rats with TBI who were treated with simvastatin [24], etanercept [2, 25], hyperbaric oxygen therapy [26], or EA [12]. Similarly, in the current study, we found numerous Caspase-3- and TUNEL-positive neurons in the ischemic cortex of TBI animals; these were significantly reduced in the EA treatment group, suggesting that EA treatment alleviates neuronal apoptosis. Based on these results, we propose that TNF-α is produced by activated microglia and astrocytes after TBI, thus activating the neuronal apoptosis pathway, and that these adverse effects could be attenuated by EA treatment [27].

Besides affecting glial TNF-α expression, as shown in this study, EA has multiple other effects in several animal models. For example, EA activates the α7 nicotinic acetylcholine receptors to attenuate inflammatory processes, thereby providing protection against cerebral ischemic injury [6]. EA also increases brain-derived neurotrophic factor expression in heat stroke [28], modulates the NF-E2 related factor 2/antioxidant response element pathway to provide protection against endotoxic shock-induced acute lung injury [29], and inhibits the ERK1/2-Egr-1 signaling pathway, thereby protecting cardiomyocytes in a mouse model of myocardial ischemia − reperfusion [30]. Thus, we believe that EA therapy may be useful for patients with TBI because of these effects. We suggest that application of EA in the acute stage of TBI may have clinical benefits.

Silver [31] demonstrated that, after TBI, glial scar formation, particularly those involving astrocytes, interfered with functional neuronal regeneration. In the present study, the TBI-induced astrogliosis was significantly attenuated by EA therapy at 72 h after TBI. Therefore, we propose that EA may have beneficial effects on neuronal regeneration.

In order to avoid interference of the effects of different treatments, recent acupuncture studies have used a sham acupuncture group as the control against which to compare the results of the experimental group. The non-acupoints are usually situated adjacent to actual acupoints, and in several experimental animal models, they have been separated by a distance of <5 mm [29, 32, 33] or have been far away from the actual acupoints [34]. For example, Zhang et al. showed that the therapeutic effects of EA applied to actual acupoints on TNF-α expression were better than the effects of EA administered at non-acupoints in a Wistar rat abdominal adhesion model [32], and Yu et al. reported the same effects in an endotoxic shock-related lung injury model in rabbits [29]. Furthermore, in SD rat models equivalent to those used in our study, Du et al. demonstrated the same results in an abdominal adhesion model even though the rats’ weights were less than those in our study [33]. Finally, Eshkevari et al. described the same results in a cold stress model in rats with weights similar to those in our study [34]. Therefore, our study did not include a non-acupoint group, but focused on comparing whether EA delivered at acupoints could notably improve the injured cortex after TBI.

Some limitations of the current study should be considered. First, only male rats were investigated. Future studies should evaluate whether EA protects female rats from TBI-induced neurobehavioral and pathological changes. Second, only one method (the inclined plane test) was used to evaluate functional outcomes, due to limited equipment availability. Third, we were unable to characterize changes in the injured brain that occurred on each day within the 3-day EA treatment window after TBI. Therefore, a time-series imaging study using this experimental TBI model/EA treatment paradigm should be conducted in future. Fourth, we did not perform EA at non-acupoints. Results from an appropriate control groups are required to clarify the specific effects of EA stimulation of acupoints and other influences.

## Conclusions

Electroacupuncture delivered for 60 min daily for 3 consecutive days ameliorates TBI in the acute stage in a rat model, by attenuating TNF-α expression in activated microglia and astrocytes and reducing neuronal apoptosis, thus contributing to improved functional outcomes. Therefore, EA may be a promising treatment strategy for TBI.

## Abbreviations

EA: Electroacupuncture
Nrf2/ARE: NF-E2 related factor 2/antioxidant response element.
PBS: Phosphate-buffered saline
SD rats: Sprague-Dawley rats
TBI: Traumatic brain injury
TNF-α: Tumor necrosis factor-alpha
α7nAChR: α7 nicotinic acetylcholine receptors
β-EP: β-endorphin
